# Supplementary material for: Exclusive Breastfeeding Rates and Associated Factors in 13 “Economic Community of West African States” (ECOWAS) Countries
Source: Nutrients. 2019 Dec 9;11(12):3007. doi: 10.3390/nu11123007 (PMC6950341; doi:10.3390/nu11123007)
Supplement: Supplementary file 1 [file nutrients-11-03007-s001.pdf]

Supplementary table S1: Adjusted OR (95% CI) of factors associated with Exclusive breastfeeding among infant aged 0-5 months in Benin, Burkina Faso, Côte d'Ivoire, Gambia, Ghana, Guinea, and Liberia.

	Benin		Burkina Faso		Côte d'Ivoire		Gambia		Ghana		Guinea		Liberia	
Characteristics	uOR(95% CI)	P-Value	uOR(95% CI)	P-Value	uOR(95% CI)	P-Value	uOR(95% CI)	P-Value	uOR(95% CI)	P-Value	uOR(95% CI)	P-Value	uOR(95% CI)	P-Value
Demographic factors														
**Residence**														
Urban	1.00		1.00		1.00		1.00		1.00		1.00		1.00	
Rural	1.24(0.91, 1.69)	0.166	2.14(1.27, 3.62)	0.005	2.03(0.84, 4.88)	0.115	1.79(0.96, 3.35)	0.068	0.53(0.26, 1.1)	0.089	0.88(0.39, 1.99)	0.765	1.28(0.68, 2.41)	0.448
**Mother's age**														
15-19 years	1.00		1.00		1.00		1.00		1.00		1.00		1.00	
20-34 years	1.12(0.79, 1.58)	0.533	0.98(0.52, 1.84)	0.954	0.96(0.38, 2.40)	0.923	1.41(0.66, 2.99)	0.373	3.77(1.12, 12.72)	0.032	1.25(0.56, 2.77)	0.585	1.12(0.42, 2.93)	0.823
35-49 years	1.06(0.62, 1.82)	0.820	0.82(0.38, 1.75)	0.608	0.64(0.16, 2.47)	0.513	1.62(0.56, 4.70)	0.373	3.27(0.81, 13.13)	0.095	0.55(0.17, 1.81)	0.324	1.33(0.40, 4.46)	0.645
**Marital status**														
Currently married	1.00		1.00		1.00		1.00		1.00		1.00		1.00	
Formerly married (div/sep/widow)	0.7(0.25, 1.96)	0.497	2.98(1.04, 8.56)	0.042	1.44(0.28, 7.29)	0.659	2.37(0.51, 10.97)	0.269	0.47(0.04, 5.24)	0.538	0.49(0.05, 4.47)	0.529	0.56(0.14, 2.20)	0.405
Never married	1.87(0.91, 3.82)	0.088	0.43(0.13, 1.42)	0.167	0.92(0.44, 1.92)	0.825	2.23(0.71, 6.99)	0.167	0.48(0.19, 1.22)	0.124	3.57(1.32, 9.69)	0.013	0.73(0.33, 1.61)	0.429
**Combined Birth rank and birth interval**														
2nd/3rd birth rank, more than 2 years interval	1.00		1.00		1.00		1.00		1.00		1.00		1.00	
1st birth rank	1.19(0.80, 1.76)	0.384	0.72(0.42, 1.22)	0.218	0.89(0.38, 2.10)	0.792	1.11(0.60, 2.02)	0.744	0.79(0.38, 1.64)	0.529	0.82(0.35, 1.90)	0.635	0.56(0.21, 1.51)	0.253
2nd/3rd birth rank, less than or equal to 2 years interval	0.84(0.51, 1.38)	0.491	1.91(0.93, 3.94)	0.079	0.44(0.12, 1.60)	0.211	0.82(0.37, 1.81)	0.625	0.39(0.17, 0.9)	0.027	*****		0.51(0.19, 1.34)	0.170
4th birth rank, more than 2 years interval	0.83(0.55, 1.24)	0.361	1.18(0.79, 1.77)	0.416	0.91(0.44, 1.90)	0.807	1.14(0.65, 2.00)	0.643	0.93(0.41, 2.11)	0.854	0.79(0.40, 1.55)	0.488	0.80(0.34, 1.86)	0.603
4th birth rank, less than or equal to 2 years interval	1.07(0.42, 2.70)	0.887	1.27(0.53, 3.07)	0.591	1.94(0.25, 14.83)	0.524	1.50(0.42, 5.30)	0.530	1.89(0.49, 7.33)	0.355	0.31(0.03, 3.34)	0.334	0.34(0.10, 1.13)	0.078
**Sex of baby**														
Male	1.00		1.00		1.00		1.00		1.00		1.00		1.00	
Female	1.41(1.09, 1.83)	0.009	0.96(0.72, 1.29)	0.796	1.58(0.88, 2.82)	0.122	1.63(1.00, 2.68)	0.052	1(0.61, 1.62)	0.986	0.95(0.61, 1.49)	0.835	1.37(0.83, 2.24)	0.214
**child Age (in months)**	0.61(0.55, 0.66)	<0.001	0.65(0.59, 0.71)	0.000	0.80(0.65, 0.97)	0.025	0.51(0.44, 0.59)	<0.001	0.6(0.52, 0.69)	<0.001	0.57(0.47, 0.69)	<0.001	0.57(0.48, 0.68)	<0.001
**Size of the baby**														

Small	1.00		1.00		1.00		1.00		1.00		1.00		1.00	
Average	0.89(0.60, 1.33)	0.576	0.94(0.60, 1.47)	0.784	1.47(0.61, 3.50)	0.389	1.87(0.99, 3.52)	0.053	2.18(1.09, 4.33)	0.027	0.57(0.27, 1.19)	0.134	1.06(0.54, 2.11)	0.859
Large	0.87(0.57, 1.31)	0.491	1.22(0.74, 2.01)	0.444	1.02(0.47, 2.21)	0.958	1.99(1.12, 3.55)	0.020	1.37(0.71, 2.65)	0.344	0.47(0.23, 0.97)	0.042	1.37(0.75, 2.50)	0.312
Socio-economic factors														
**Household Wealth Index**														
Poorest	1.00		1.00		1.00		1.00		1.00		1.00		1.00	
Poorer	1.01(0.65, 1.56)	0.965	0.77(0.47, 1.25)	0.288	0.79(0.30, 2.10)	0.634	0.89(0.51, 1.56)	0.679	0.36(0.15, 0.87)	0.024	0.94(0.33, 2.70)	0.908	1.47(0.76, 2.82)	0.247
Middle	0.93(0.62, 1.4)	0.718	0.79(0.51, 1.23)	0.302	0.84(0.36, 1.94)	0.678	0.61(0.35, 1.06)	0.080	0.31(0.13, 0.74)	0.009	0.49(0.22, 1.06)	0.069	1.40(0.69, 2.85)	0.352
Richer	1.26(0.80, 1.99)	0.309	1.08(0.69, 1.68)	0.750	1.07(0.37, 3.09)	0.898	0.91(0.43, 1.96)	0.812	0.18(0.06, 0.53)	0.002	0.76(0.34, 1.72)	0.510	0.68(0.25, 1.88)	0.460
Richest	0.95(0.57, 1.61)	0.856	0.73(0.38, 1.39)	0.333	3.02(0.99, 9.27)	0.053	1.08(0.42, 2.80)	0.868	0.08(0.02, 0.28)	<0.001	0.44(0.14, 1.38)	0.157	0.72(0.22, 2.34)	0.580
**Work in the last 12 months**														
Not working	1.00		1.00		1.00		1.00		1.00		1.00		1.00	
Working	1.24(0.90, 1.69)	0.183	0.94(0.67, 1.32)	0.715	0.71(0.40, 1.26)	0.244	1.01(0.60, 1.69)	0.969	0.83(0.49, 1.39)	0.475	0.85(0.50, 1.44)	0.542	1.13(0.68, 1.89)	0.625
**Maternal education**														
No education	1.00		1.00		1.00		1.00		1.00		1.00		1.00	
Primary	1.05(0.70, 1.56)	0.829	1.4(0.88, 2.21)	0.155	0.89(0.35, 2.28)	0.814	0.95(0.54, 1.68)	0.863	0.55(0.24, 1.24)	0.146	0.64(0.30, 1.36)	0.241	1.18(0.59, 2.37)	0.640
Secondary and above	1.08(0.61, 1.91)	0.785	2.28(0.75, 6.9)	0.146	0.75(0.18, 3.04)	0.683	0.68(0.16, 2.94)	0.607	1.38(0.62, 3.09)	0.433	0.71(0.06, 7.88)	0.776	1.01(0.18, 5.67)	0.994
**Maternal Literacy**														
Cannot read at all	1.00		1.00		1.00		1.00		1.00		1.00		1.00	
Able to read only part of sentences	0.87(0.51, 1.48)	0.609	1.08(0.37, 3.17)	0.891	1.41(0.46, 4.33)	0.549	2.77(0.64, 11.99)	0.173	1.54(0.71, 3.36)	0.273	1.26(0.13, 12.45)	0.845	2.18(0.45, 10.58)	0.333
Access to media														
**Frequency of reading newspaper or magazine**														
Not at all	1.00		1.00		1.00		1.00		1.00		1.00		1.00	
Less than once a week	1.35(0.55, 3.27)	0.512	1.17(0.35, 3.91)	0.794	1.39(0.44, 4.39)	0.571	0.91(0.38, 2.16)	0.834	0.62(0.25, 1.55)	0.308	0.11(0.02, 0.58)	0.009	0.6(0.15, 2.33)	0.460
At least once a week/ Almost every day	1.58(0.57, 4.39)	0.384	1.59(0.45, 5.60)	0.473	2.18(0.67, 7.06)	0.193	0.36(0.10, 1.34)	0.127	4.61(1.16, 18.27)	0.030	1.21(0.22, 6.71)	0.827	0.39(0.13, 1.2)	0.100
**Frequency of listening to Radio**														
Not at all	1.00		1.00		1.00		1.00		1.00		1.00		1.00	
Less than once a week	0.55(0.38, 0.79)	0.001	0.74(0.51, 1.08)	0.119	0.60(0.23, 1.55)	0.286	0.91(0.42, 1.99)	0.821	1.62(0.75, 3.52)	0.221	1.30(0.65, 2.62)	0.455	1.03(0.56, 1.91)	0.927
At least once a week/ Almost every day	0.64(0.46, 0.89)	0.007	0.80(0.56, 1.15)	0.228	1.49(0.73, 3.04)	0.269	1.78(0.93, 3.39)	0.080	1.56(0.73, 3.35)	0.248	1.31(0.68, 2.53)	0.422	1.06(0.49, 2.30)	0.873
**Frequency of watching Television**														

Not at all	1.00		1.00		1.00		1.00		1.00		1.00		1.00	
Less than once a week	0.87(0.59, 1.29)	0.488	1.89(1.17, 3.05)	0.009	1.30(0.52, 3.26)	0.581	0.82(0.42, 1.61)	0.561	1.39(0.67, 2.89)	0.372	0.88(0.41, 1.90)	0.752	0.64(0.29, 1.41)	0.268
At least once a week/ Almost every day	1.10(0.67, 1.81)	0.712	2.64(1.61, 4.33)	<0.001	0.56(0.24, 1.31)	0.181	0.54(0.29, 0.99)	0.047	1.37(0.65, 2.89)	0.404	1.90(0.84, 4.28)	0.121	0.36(0.13, 1.02)	0.054
Healthcare utilization factors														
**Place of delivery**														
Home	1.00		1.00		1.00		1.00		1.00		1.00		1.00	
Health facility	1.72(1.03, 2.89)	0.040	0.58(0.25, 1.31)	0.189	15.52(4.28, 56.34)	<0.001	0.99(0.26, 3.80)	0.989	0.26(0.08, 0.82)	0.022	0.78(0.34, 1.79)	0.552	0.86(0.26, 2.81)	0.803
**Mode of delivery**														
Non-caesarean	1.00		1.00		1.00		1.00		1.00		1.00		1.00	
Caesarean section	0.88(0.39, 1.98)	0.757	0.64(0.25, 1.66)	0.360	3.62(1.13, 11.64)	0.031	1.43(0.43, 4.78)	0.556	1.09(0.49, 2.43)	0.833	0.34(0.08, 1.34)	0.123	1.98(0.54, 7.29)	0.305
**Type of delivery assistance**														
Health professional	1.00		1.00		1.00		1.00		1.00		1.00		1.00	
Traditional birth attendant.	*****		0.44(0.21, 0.95)	0.037	*****		*****		0.15(0.03, 0.83)	0.029	*****		*****	
Other untrained	0.89(0.49, 1.60)	0.687	1.22(0.07, 21.74)	0.892	6.12(1.67, 22.5)	0.007	1.08(0.25, 4.67)	0.923	*****		0.45(0.18, 1.12)	0.086	1.43(0.39, 5.25)	0.587
No one	1.38(0.78, 2.44)	0.271	0.73(0.13, 4.03)	0.719	2.93(0.25, 34.1)	0.390	0.81(0.14, 4.78)	0.812	0.25(0.05, 1.20)	0.082	0.22(0.06, 0.82)	0.024	*****	
**Antenatal Clinic visits**														
None	1.00		1.00		1.00		1.00		1.00		1.00		1.00	
1--3	0.86(0.52, 1.43)	0.567	1.71(0.66, 4.42)	0.266	0.66(0.17, 2.52)	0.546	1.47(0.12, 17.43)	0.761	6.35(1.18, 34.24)	0.032	0.92(0.31, 2.72)	0.878	1.46(0.16, 13.35)	0.736
4+	0.81(0.49, 1.34)	0.409	2.24(0.82, 6.13)	0.116	0.87(0.20, 3.78)	0.848	1.94(0.16, 23.72)	0.603	2.04(0.41, 10.26)	0.384	0.63(0.22, 1.78)	0.383	1.56(0.16, 14.74)	0.699

***** no estimate due to small or empty cell

Supplementary table S2: Adjusted OR (95% CI) of factors associated with Exclusive breastfeeding among infant aged 0-5 months in Mali, Niger, Nigeria, Senegal, Sierra Leone, and Togo.

Characteristics	Mali uOR(95% CI)	Mali P-Value	Niger uOR(95% CI)	Niger P-Value	Nigeria uOR(95% CI)	Nigeria P-Value	Senegal uOR(95% CI)	Senegal P-Value	Sierra Leone uOR(95% CI)	Sierra Leone P-Value	Togo uOR(95% CI)	Togo P-Value
Demographic factors												
**Residence**												
Urban	1.00		1.00		1.00		1.00		1.00		1.00	
Rural	2.41(1.3, 4.45)	0.005	1.41(0.64, 3.11)	0.396	0.85(0.59, 1.23)	0.392	0.92(0.61, 1.40)	0.702	1.19(0.58, 2.46)	0.628	1.03(0.31, 3.40)	0.965
**Mother's age**												
15-19 years	1.00		1.00		1.00		1.00		1.00		1.00	
20-34 years	1.80(1.01, 3.23)	0.048	0.80(0.42, 1.51)	0.483	1.44(0.74, 2.79)	0.278	1.65(0.86, 3.17)	0.134	0.89(0.44, 1.78)	0.736	1.30(0.54, 3.16)	0.560
35-49 years	2.59(1.14, 5.91)	0.023	0.85(0.38, 1.94)	0.702	1.86(0.86, 4.01)	0.114	2.37(0.98, 5.75)	0.056	1.19(0.41, 3.43)	0.753	1.39(0.49, 3.95)	0.530
**Marital status**												
Currently married	1.00		1.00		1.00		1.00		1.00		1.00	
Formerly married (div/sep/widow)	2.10(0.30, 14.8)	0.456	*****		1.07(0.45, 2.54)	0.885	0.59(0.17, 2.07)	0.407	0.77(0.18, 3.24)	0.718	0.30(0.03, 3.03)	0.304
Never married	1.22(0.39, 3.75)	0.734	9.24(0.7, 122.91)	0.092	0.49(0.23, 1.03)	0.059	0.67(0.23, 1.96)	0.465	0.54(0.27, 1.10)	0.088	0.91(0.26, 3.17)	0.877
**Combined Birth rank and birth interval**												
2nd/3rd birth rank, more than 2 years interval	1.00		1.00		1.00		1.00		1.00		1.00	
1st birth rank	1.65(0.87, 3.12)	0.123	0.71(0.38, 1.33)	0.284	1.08(0.71, 1.64)	0.733	1.49(0.92, 2.40)	0.102	0.96(0.53, 1.76)	0.905	0.63(0.35, 1.13)	0.118
2nd/3rd birth rank, less than or equal to 2 years interval	1.88(0.95, 3.72)	0.071	0.81(0.42, 1.55)	0.516	1.14(0.73, 1.78)	0.567	1.46(0.75, 2.81)	0.262	0.54(0.22, 1.32)	0.176	0.51(0.17, 1.50)	0.223
4th birth rank, more than 2 years interval	0.86(0.55, 1.35)	0.514	0.84(0.48, 1.46)	0.529	0.72(0.48, 1.08)	0.113	1.04(0.64, 1.69)	0.882	1.04(0.59, 1.85)	0.884	0.71(0.37, 1.37)	0.306
4th birth rank, less than or equal to 2 years interval	0.86(0.46, 1.60)	0.629	0.72(0.35, 1.47)	0.368	0.46(0.22, 1.00)	0.051	1.06(0.45, 2.51)	0.888	0.71(0.27, 1.87)	0.484	0.78(0.28, 2.19)	0.639
**Sex of baby**												
Male	1.00		1.00		1.00		1.00		1.00		1.00	
Female	1.01(0.68, 1.50)	0.972	1.18(0.82, 1.70)	0.365	1.37(1.03, 1.81)	0.028	1.02(0.74, 1.41)	0.890	0.88(0.61, 1.25)	0.462	1.54(1.01, 2.33)	0.044
**child Age (in months)**	0.62(0.55, 0.69)	<0.001	0.76(0.68, 0.86)	<0.001	0.72(0.66, 0.79)	<0.001	0.63(0.56, 0.70)	<0.001	0.76(0.67, 0.86)	<0.001	0.67(0.58, 0.78)	<0.001
**Size of the baby**												

Small	1.00		1.00		1.00		1.00		1.00		1.00	
Average	0.75(0.48, 1.18)	0.213	1.35(0.88, 2.09)	0.170	1.84(1.20, 2.81)	0.005	1.38(0.92, 2.07)	0.116	0.86(0.47, 1.59)	0.639	1.32(0.74, 2.37)	0.345
Large	1.04(0.64, 1.70)	0.870	1.68(1.01, 2.79)	0.047	1.87(1.20, 2.94)	0.006	1.06(0.69, 1.62)	0.795	1.01(0.55, 1.87)	0.963	1.34(0.74, 2.44)	0.332
Socio-economic factors												
**Household Wealth Index**												
Poorest	1.00		1.00		1.00		1.00		1.00		1.00	
Poorer	1.38(0.78, 2.43)	0.263	0.72(0.39, 1.33)	0.293	1.20(0.7, 2.06)	0.503	1.24(0.82, 1.87)	0.310	1.08(0.59, 1.99)	0.803	0.58(0.3, 1.14)	0.113
Middle	1.09(0.62, 1.90)	0.763	0.87(0.48, 1.58)	0.648	1.22(0.65, 2.3)	0.535	1.22(0.70, 2.13)	0.473	1.11(0.60, 2.04)	0.739	0.60(0.3, 1.19)	0.143
Richer	1.57(0.83, 2.99)	0.166	0.90(0.47, 1.72)	0.758	1.28(0.63, 2.59)	0.493	1.07(0.56, 2.06)	0.841	0.71(0.38, 1.33)	0.288	1.16(0.3, 4.46)	0.825
Richest	0.87(0.43, 1.73)	0.684	1.14(0.43, 3.01)	0.786	2.09(1.00, 4.35)	0.050	1.56(0.65, 3.73)	0.316	0.41(0.15, 1.08)	0.070	1.02(0.24, 4.4)	0.980
**Work in the last 12 months**												
Not working	1.00		1.00		1.00		1.00		1.00		1.00	
Working	1.14(0.80, 1.63)	0.473	1.40(0.87, 2.24)	0.168	1.34(0.98, 1.82)	0.066	1.07(0.75, 1.53)	0.725	0.71(0.47, 1.10)	0.123	1.00(0.63, 1.58)	1.000
**Maternal education**												
No education	1.00		1.00		1.00		1.00		1.00		1.00	
Primary	0.91(0.53, 1.57)	0.726	1.04(0.58, 1.86)	0.890	2.15(1.36, 3.38)	0.001	2.32(1.45, 3.70)	<0.001	0.84(0.47, 1.51)	0.551	1.87(1.09, 3.21)	0.024
Secondary and above	0.67(0.33, 1.37)	0.270	1.58(0.41, 6.17)	0.507	0.87(0.34, 2.18)	0.760	2.16(0.82, 5.68)	0.118	0.30(0.04, 2.24)	0.237	1.00(0.27, 3.73)	0.996
**Maternal Literacy**												
Cannot read at all	1.00		1.00		1.00		1.00		1.00		1.00	
Able to read only part of sentences	0.79(0.35, 1.79)	0.568	0.88(0.27, 2.92)	0.836	2.56(1.1, 5.93)	0.029	0.51(0.25, 1.05)	0.067	4.53(0.68, 30.24)	0.119	2.61(0.82, 8.35)	0.104
Access to media												
**Frequency of reading newspaper or magazine**												
Not at all	1.00		1.00		1.00		1.00		1.00		1.00	
Less than once a week	1.65(0.49, 5.52)	0.417	0.53(0.17, 1.72)	0.293	1.18(0.79, 1.78)	0.412	1.24(0.57, 2.69)	0.591	2.36(0.74, 7.58)	0.148	1.19(0.42, 3.36)	0.737
At least once a week/ Almost every day	0.85(0.23, 3.13)	0.803	0.59(0.16, 2.18)	0.424	1.03(0.64, 1.65)	0.906	1.18(0.49, 2.81)	0.714	1.35(0.54, 3.39)	0.525	2.17(0.4, 11.79)	0.367
**Frequency of listening to Radio**												
Not at all	1.00		1.00		1.00		1.00		1.00		1.00	
Less than once a week	1.20(0.72, 2.01)	0.482	1.30(0.80, 2.10)	0.290	0.89(0.55, 1.44)	0.636	1.09(0.70, 1.69)	0.694	0.66(0.35, 1.24)	0.192	0.93(0.50, 1.73)	0.819

At least once a week/ Almost every day	1.07(0.68, 1.69)	0.754	1.83(1.12, 3.00)	0.016	0.90(0.6, 1.34)	0.592	0.98(0.64, 1.51)	0.942	0.53(0.34, 0.83)	0.006	1.17(0.59, 2.33)	0.642
**Frequency of watching Television**												
Not at all	1.00		1.00		1.00		1.00		1.00		1.00	
Less than once a week	0.73(0.43, 1.23)	0.230	0.61(0.33, 1.15)	0.126	1.10(0.7, 1.74)	0.676	0.54(0.35, 0.83)	0.006	1.01(0.30, 3.37)	0.986	1.73(0.55, 5.40)	0.344
At least once a week/ Almost every day	0.94(0.61, 1.45)	0.768	0.65(0.32, 1.33)	0.242	1.19(0.8, 1.76)	0.392	0.65(0.39, 1.08)	0.098	1.72(0.78, 3.82)	0.181	0.68(0.30, 1.50)	0.334
Healthcare utilization factors												
**Place of delivery**												
Home	1.00		1.00		1.00		1.00		1.00		1.00	
Health facility	0.75(0.46, 1.24)	0.268	0.60(0.12, 2.89)	0.521	1.17(0.8, 1.70)	0.418	1.36(0.76, 2.41)	0.295	0.74(0.31, 1.74)	0.484	0.27(0.02, 3.68)	0.326
**Mode of delivery**												
Non-caesarean	1.00		1.00		1.00		1.00		1.00		1.00	
Caesarean section	0.44(0.14, 1.41)	0.168	0.57(0.17, 1.89)	0.360	0.68(0.32, 1.46)	0.323	0.46(0.22, 0.97)	0.042	1.31(0.52, 3.27)	0.567	1.45(0.63, 3.34)	0.385
**Type of delivery assistance**												
Health professional	1.00		1.00		1.00		1.00		1.00		1.00	
Traditional birth attendant.	*****		*****		*****		*****		*****		0.36(0.03, 4.76)	0.435
Other untrained	0.59(0.21, 1.61)	0.300	0.26(0.05, 1.42)	0.120	*****		0.97(0.63, 1.51)	0.901	0.96(0.38, 2.43)	0.934	0.52(0.04, 6.54)	0.611
No one	0.80(0.21, 3.07)	0.747	0.58(0.11, 3.01)	0.517	0.50(0.28, 0.90)	0.021	1.47(0.58, 3.69)	0.416	0.72(0.11, 4.58)	0.728	0.17(0.01, 2.61)	0.202
**Antenatal Clinic visits**												
None	1.00		1.00		1.00		1.00		1.00		1.00	
1--3	1.52(0.86, 2.67)	0.146	1.00(0.52, 1.94)	0.990	0.95(0.56, 1.61)	0.851	2.45(0.65, 9.14)	0.183	1.71(0.24, 12.07)	0.589	1.76(0.68, 4.51)	0.240
4+	1.00(0.54, 1.84)	0.995	1.24(0.62, 2.47)	0.542	1.34(0.84, 2.14)	0.217	2.57(0.68, 9.73)	0.163	1.46(0.20, 10.85)	0.708	1.93(0.71, 5.28)	0.199

***** no estimate due to small or empty cell
